# Primary Orbital Mesenchymal Chondrosarcoma: Case Report and Review of the Literature

**DOI:** 10.1155/2012/292147

**Published:** 2012-06-18

**Authors:** Angel Herrera, Cesar Ortega, Gervith Reyes, Miguel Angel Alvarez, Daniela Tellez

**Affiliations:** ^1^Department of Surgical Oncology, Instituto Nacional de Cancerología, Mexico City, Mexico; ^2^Department of Head and Neck Surgery, Instituto Nacional de Cancerología, Mexico City, Mexico; ^3^Department of Oncology, Instituto Nacional de Cancerología, Mexico City, Mexico

## Abstract

Orbital mesenchymal chondrosarcoma is a very uncommon lesion of the bone and extraskeletal tissue. To our knowledge, approximately 30 cases have been described. We present the case of a 52-year-old male who presented with a history of progressive proptosis and chemosis of the right eye caused by an orbital tumor. He underwent exenteration of the right orbit, and the histological examination revealed a mesenchymal orbital chondrosarcoma. This paper attempts to describe a rare entity that should be considered in the differential diagnosis of calcified orbital lesions, especially in young adults. Complete removal of the tumor is the mainstay of treatment, but adjuvant radiation therapy and chemotherapy should be considered.

## 1. Introduction

Mesenchymal chondrosarcoma of the head and neck is an uncommon tumor with potential for exhibiting highly aggressive behavior. This lesion is a malignant small round cell neoplasm with focal cartilaginous differentiation, often with a pericytomatous vascular pattern. It represents approximately 1% of all chondrosarcomas [1] and approximately 0.1% of all head and neck neoplasms [2, 3]. In the head and neck region, it presents with a predilection for the maxillofacial skeleton, where it has been reported to occur in particular in the mandible and maxilla [4].

 It is usually seen in younger age group compared to conventional chondrosarcomas [3].

There are approximately 30 cases described in the literature. The aim of this paper is to report a case of mesenchymal chondrosarcoma of the orbit.

## 2. Case Presentation

**Figure 1:**
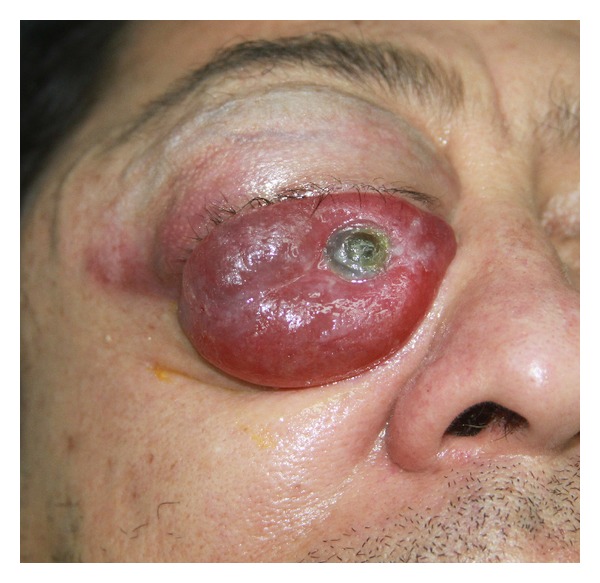
Patient at the onset with inferior displacement of the globe and severe conjunctival chemosis.

**Figure 2:**
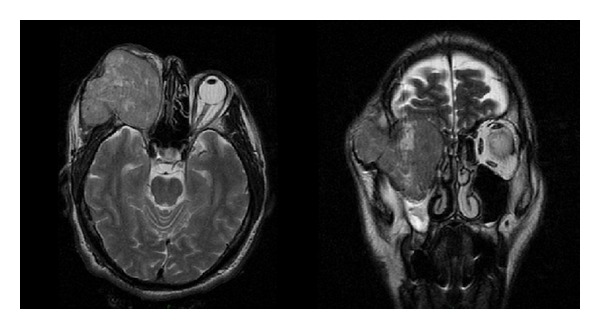
Axial and coronal MRI image showing a right intraconic and extrasonic mass with ocular globe displacement and temporal muscle and zygomatic bone infiltration.

A 52-year-old man, without any past medical illness, presented with a 12-month history of progressive proptosis of the right eye. Physical examination revealed a decreased right visual acuity, inferior displacement of the globe, severe conjunctival chemosis, and reduced corneal sensitivity (Figure 1). No abnormality of the left eye was detected. An incisional biopsy was performed at a different institution, and the pathology report demonstrated a fusocellular neoplasm with hemangiopericytoma pattern, also compatible with monophasic synovial sarcoma. Computed tomography (CT) and magnetic resonance imaging (MRI) scans revealed a well-defined right intrasonic mass, measuring 5.3 × 4.6 × 3.6 cm, with ocular globe displacement but without involvement of the temporal fossa (Figure 2). Upon admission, the patient had a Karnofsky score of 90% and was classified as grade zero according to the status of the Eastern Cooperative Oncology Group (ECOG).

**Figure 3:**
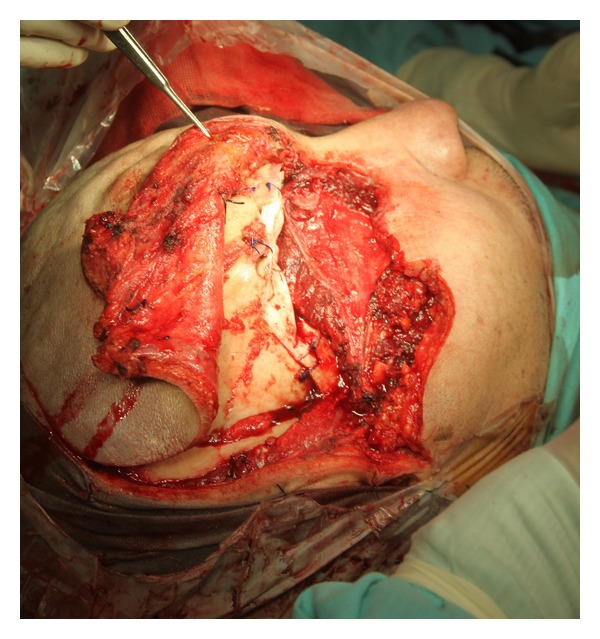
Orbitozygomatic approach. Tumoral resection and reconstruction with cyanoacrylate.

**Figure 4:**
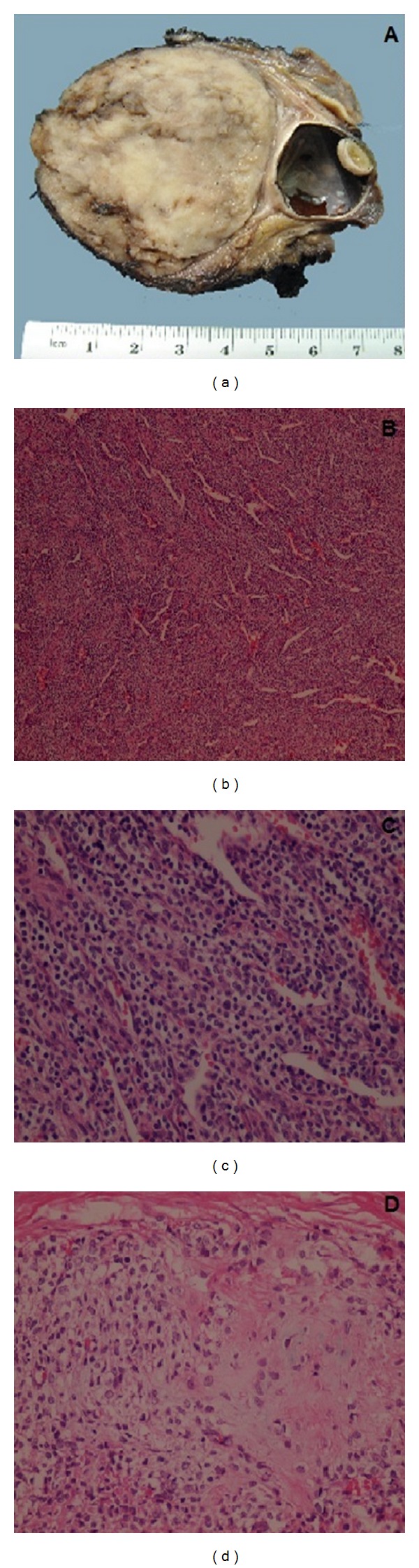
(a) Macroscopic view of the resected specimen. (b) Microphotography (4x) of the neoplasm showing a hemangiopericytoma-like vascular pattern, with proliferation of small, round cells with clear cytoplasm. (c) Closer view of the previous microphotography (40x), showing the small round cells surrounding the vessels. (d) Microphotography (40x) showing the transition between well differentiated cartilage and small cell areas.

Based on this diagnosis, a right lateral orbital exenteration with excision of the mass by a craniofacial approach was performed. We found a mass that measured 7 × 8 cm with sphenoidal fissure obliteration and destruction of the roof and lateral wall of the orbit. Based on these findings, the lesion was resected, and we performed an orbital roof plasty with cyanoacrylate (Figure 3). There were no postoperative complications. Definitive histopathological examination revealed a mesenchymal chondrosarcoma located in the orbital soft tissue, with temporal muscle and zygomatic bone infiltration and a 5 mm bone margin. The posterior edge of the ocular globe, the globe itself, and the optic nerve did not show evidence of the neoplasm (Figure 4).

The patient was advised adjuvant chemotherapy and radiotherapy. He received a total of 66 Gy of external radiotherapy to the temporozygomatic region, showing no signs of toxicity or infection. He is currently on a regimen of vincristine, adriamycin, and cyclophosphamide alternating with ifosfamide and etoposide (VAC/IE), which is used in the treatment of patients with Ewing's Sarcoma. Currently, the patient is alive and without evidence of disease.

## 3. Discussion

The first case of mesenchymal chondrosarcoma (MCS) was described by Lichtenstein and Bernstein in 1959 [5]. Approximately 10% occur in the head and neck region, being the maxillomandibulofacial skeleton the site in the head and neck where mesenchymal chondrosarcomas are most commonly encountered. Less commonly encountered sites in the head and neck include the sinonasal tract, orbit, and thyroid gland [1]. Our case is one of the approximately 30 cases already reported with localization in the orbit. This tumor tends to affect patients in their second or third decades of life, with a female preponderance [1, 6], something important to notice given that the patient herein described is a 52-year-old male, in contrast with the typical sex and age of onset.

Clinically, these tumors tend to present with compression of the ocular globe resulting in progressive proptosis and/or visual abnormalities, such as diplopia or reduction in visual acuity. The radiological examination is relevant to help establish the diagnosis. CT scans usually reveal presence of foci of calcification in the lesion. MRI scans are helpful to delimitate the lesion margins [7]. Our patient presented these clinical and radiological findings, in accordance with previous reports in the literature.

Histologically, this tumour exhibits a biomorphic appearance of undifferentiated mesenchymal cells with islands of mature hyaline cartilage [8]. Immunohistochemical analysis often reveals positivity for vimentin, S100 protein and CD99; meanwhile, actin, cytokeratin, and EMA are typically negative [9, 10]. The differential diagnosis of orbital MCS includes hemangiopericytoma, myxochondrosarcoma, osteogenic sarcoma, and osteochondroma as primary tumors and lymphoma, neuroblastoma, synovial cell sarcoma, and chondrosarcoma as tumors affecting the orbit secondarily (direct invasion or metastasis) [11]. This case presented no difficulty in the histopathological diagnosis since it showed a characteristic morphological pattern composed of round cells with an abrupt transition to well-differentiated hyaline cartilage and a hemangiopericytoma-like vascular pattern. The immunohistochemical analysis was positive for S100 protein and negative for cytokeratin and actin, which are some of the immunohistochemical characteristics often described for this type of tumors. In this case, hemangiopericytoma was considered the principal differential diagnosis.

Surgical resection with wide margins is known as the most effective treatment modality for chondrosarcoma [3]. Zakkak et al. reviewed various treatment modalities in the maxilla and mandible and found that the best outcome was after radical surgery [12]. The overall low incidence of regional metastasis may suggest that neck dissection is not indicated.

Radiotherapy might play a role when accompanied by surgery although some believe MCS to be a radioresistant tumor [3, 13]. Some authors report the use of preoperative radiation therapy to reduce tumor bulk prior to radical resection, with the hope of reducing extension in continuity or by micrometastasis, but it does not change the preoperative approach. Postoperative radiotherapy does not show a statistically significant proof of better prognosis even when there is evidence that demonstrates trend toward increased survival [14].

Chemotherapy has a limited role in chondrosarcoma and should be used as an adjuvant therapy in cases with aggressive behavior with potential metastasis, rapid local recurrence, and high-grade lesions [2, 3, 13]. This patient was advised the same chemotherapy regimen as used for Ewing's sarcoma. The standard neoadjuvant/adjuvant chemotherapy backbone consists of vincristine, dactinomycin, cyclophosphamide, and doxorubicin (VACD). Since then, a number of studies have sought to improve on that standard, using chemotherapy with VACD either alone or alternating with ifosfamide and etoposide (VACD-IE), the latter significantly improved 5-year disease-free survival (DFS) (69% versus 54%, *P* = .005), and mesenchymal chondrosarcoma could be treated as per Ewing's sarcoma [15].

There are numerous active signaling pathways that have been described in human chondrosarcoma, trying to suggest potential molecular targets for directed chemotherapy. One of these is a pathway dependent on phosphoinositide-3 kinase and MEK-extracellular signal-regulated kinase (ERK) signaling. Additionally, chondrosarcoma cell proliferation and degradation is dependent on peroxisome proliferator-activated receptor-gamma (PPAR-c) activity, with a loss of PPAR-c expression and associated apoptosis in high-grade tumors. Targeting these pathways may improve control of cranial chondrosarcoma and decrease the need of recurrent operations [16].

The prognosis of patients with MCS is extremely variable, ranging from complete tumor response and long-term survival, to rapid local tumor progression with widespread metastasis. The mesenchymal histology alone carries a nearly 10-fold increase in 5-year mortality. The overall 5- and 10-year survival for patients with mesenchymal chondrosarcoma, when considering all sites, is 55% and 27%, respectively. It has been postulated that earlier detection and diagnosis of lesions in the head and neck may in part account for improved survival for mesenchymal chondrosarcoma located in the head and neck [1, 17].

## 4. Conclusion

Orbital mesenchymal chondrosarcoma is a rare entity and should be considered in the differential diagnosis of calcified orbital lesions, especially in young adults. Complete removal of the tumor, either by orbital resection or exenteration, is the mainstay of treatment. Adjuvant radiation therapy and chemotherapy should be considered, due to the histologically aggressive nature of the tumor.
